# The Relation between Teachers’ and Children’s Playfulness: A Pilot Study

**DOI:** 10.3389/fpsyg.2017.02214

**Published:** 2017-12-19

**Authors:** Shulamit Pinchover

**Affiliations:** The Paul Baerwald School of Social Work and Social Welfare, Hebrew University of Jerusalem, Jerusalem, Israel

**Keywords:** playfulness, teachers, children, early childhood, teacher–child interaction

## Abstract

Young children spend considerable time in educational settings, in which traditionally, their primary occupation is play. A playful preschool environment has been related to better cognitive, social and emotional development. Although it is assumed that teachers’ playful behaviors are important in creating a playful school environment, empirical knowledge on this subject is lacking. The current study pilot examines the relation between teachers’ and children’s playfulness. Thirty-one teacher–child dyads participated. The teachers were asked to complete the Adult Playfulness Scale (APS). Thirty-minute videotapes of teacher–child play interactions were used to evaluate the child’s playfulness using the Test of Playfulness. A positive relation was found between two of the APS subscales (spontaneity and silliness) and child playfulness. Teacher silliness mediated the relation between children’s age and playfulness. This study is the first to show that teachers’ playfulness aspects are related to higher playfulness in children. Promoting teachers’ playful behaviors can be related to better teacher–child playful interactions, thereby enhancing children’s playfulness.

## Introduction

Young children spend considerable time in educational settings, in which traditionally, their primary occupation is play (Wong and Logan, 2016; Pyle et al., 2017). Play and playfulness are considered basic features of early childhood education (ECE) and have been related to social, emotional, and cognitive development (Fisher, 1992; Frost et al., 2001; Youell, 2008). A playful school environment has been related to higher child involvement in play, and to improved learning and development (Jones and Reynolds, 2015). However, despite the extensive literature on how to create a playful educational environment (Jones and Reynolds, 2015), and the common intuitive assumption regarding the importance of teachers’ playful behaviors in promoting a playful climate in class and developing children’s play and playfulness, empirical knowledge on the subject is lacking (Singer, 2013). First, adults’ and specifically teachers’ playfulness is less studied compared to children’s (Proyer, 2012). Additionally, the relationship between teachers’ and children’s playfulness has hardly been examined empirically, to the best of our knowledge.

The current study is part of a broader research project on play interactions between children and adults at home and in educational settings. Specifically, it aims to examine the relation between aspects of teachers’ perceived playfulness (including spontaneous, expressive, fun, creative, and silly behaviors) and children’s observed playfulness in ECE settings.

### Play in Early Childhood Education

Over the years, several attempts have been made to understand the role of play in ECE, based on extensive theoretical and empirical literature supporting its significance for young children’s development and well-being (for a review, see Johnson et al., 2013). There is substantial evidence that through play and playfulness children demonstrate improved verbal and social communication, high levels of interaction skills, creativity, imagination, divergent thinking, and problem-solving skills (see review at Wood and Attfield, 2005). Playful educational environment was found to foster creativity and imagination along with academic achievements (Kangas, 2010; Kangas and Ruokamo, 2012). Specifically, playful ECE settings are considered helpful in developing young children’s play. Although it is almost axiomatic that play is a cornerstone in ECE (Pyle et al., 2017), in recent years it seems that play has been sidelined by early learning standards and assessments of academic attainments (Roskos and Christie, 2007; Bodrova, 2008; Wisneski and Reifel, 2012; Dickey et al., 2016). This is increasingly so as the children grow older. This trend, at least in the developed world, is so acute, in fact, that lately play interventions have been increasingly attempted in order to teach teachers and children to create a playful and imaginative world together (Lobman, 2003, 2006).

The teacher is considered to play a significant role in creating a playful environment and developing children’s play (Pramling Samuelsson and Johansson, 2009; Johnson et al., 2013). Teachers’ role includes planning of the setting for play, using a playful pedagogic approach, and engaging with children in play (Wood, 2008). Teachers’ involvement in play interactions can increase the frequency, duration, and complexity of children’s play (McAfee and Leong, 2010). Vygotsky (1978) emphasizes the active role teachers have in children’s play. However, there is a debate on how the teacher should be involved: as a co-player, an instructor or a supervisor (Jones and Reynolds, 2015). Most scholars would agree that teachers need to create a playful classroom environment and allow spontaneous child–child play activity, but also be skillful play partners themselves (Ashiabi, 2007; Childress, 2010). For example, a study with Dutch teachers found that teachers’ higher involvement in play interaction is related to children’s higher involvement in play (Singer et al., 2014). In real life, however, teachers are often too busy with classroom management to be available to promote play, or simply do not know how to do that (Aras, 2016; Elliott and Jarneman, 2017).

### Playfulness

Playfulness is defined as the disposition to engage in play (Barnett, 1991), and is considered a personality trait that exists and is expressed across the lifespan (Lieberman, 1977). According to Lieberman (1977), each child or person has a different playfulness style, affected by personality as well as environmental characteristics, such as those of home and educational setting. Proyer (2012) found it to have significant role in both children and adults’ lives. Recent studies have examined the lifetime flexibility of playfulness and the ability to enhance it using various interventions (Bundy et al., 2008). Results indicate that it is responsive to intervention and can be change over time (Okimoto et al., 2000; Case-Smith, 2013; Fabrizi, 2014).

#### Children’s Playfulness

The way children play can be captured in different ways; one of them is through playfulness (Bundy, 1997; Cornelli Sanderson, 2010). Playfulness not only captures the mechanism of play, but also addresses the child’s general approach to play (Bundy, 1997). Playfulness is composed of four dimensions: (1) the child’s internal motivation independently of external expectations; (2) internal control – the child’s ability to determine or direct the play action; (3) the freedom to suspend reality in play; and (4) framing – the child’s ability to communicate and interpret social cues. Playfulness has been related to children’s social, emotional and cognitive development and well-being (Youell, 2008). For example, it is significantly related to active coping, affective regulation, and willingness to express emotions (Christian, 2012).

When children play, they learn about reality and ways of affecting and manipulating it. Being playful means being free to create roles and activities, regardless of external constraints. Children’s playful behavior is guided by an internal motivation for a process with self-imposed goals, with a tendency to attribute their own meanings to objects and behaviors (Rubin et al., 1983). Those characteristics of playfulness help children learn, be creative and cope with difficulties (Youell, 2008).

#### Teachers’ Playfulness

While the literature on adults’ playfulness has been growing in recent years, it is still understudied compared to children’s playfulness (Proyer, 2012). Individuals who are playful are typically funny, humorous, spontaneous, and are more likely to act in a playful manner by joking, teasing, clowning, and being silly. Existing studies have shown that adults’ playfulness is related to well-being, sense of happiness, relationship satisfaction, and higher self-estimates of ingenuity and creativity (Proyer, 2012, 2013, 2014; Bateson et al., 2013; Yue et al., 2016). Specifically, research indicates that playfulness is positively related to both job satisfaction and job performance (Yu et al., 2007). Two recent studies have focused on parental playfulness. A study from 32 young adult’s perspective reported a positive relation between parents’ playfulness and children’s adoptive behavior (Shen et al., 2017). Similarly, Menshe-Grinberg and Atzaba-Poria (2017) found that parental playfulness moderate the relation between parental behaviors and child’s negativity.

However, the playfulness of educational professionals has rarely been investigated. Based on a small exploratory study of 16 teachers (Lieberman, 1974), and on other qualitative research observations in a natural environment, Lieberman (1977) emphasized the importance of teachers’ playfulness in relation to children’s play, playfulness and divergent thinking. She concluded that through playfulness behaviors, the teacher could create an environment that enables children to express greater joy and be more creative and flexible in play. The few other studies available show that teacher playfulness is related to teacher–child interaction and relationship (Graham et al., 1989; Tegano et al., 1999; McMillan, 2017). For example, a qualitative case study of two ECE teachers showed that teachers’ playful behavior could alleviate toddlers’ emotional distress and help children in transitions and that playfulness is a constructive way to build a secure relationship between toddlers and teachers (Jung, 2011). Another study examined Lithuanian and Greek teachers’ perceptions regarding playfulness in their kindergarten class and found that different teacher found different ways to engage in playful behaviors in class. While teachers in Lithuania believed that their role as adults was to promote playfulness and a playful atmosphere, Greek teachers paid more attention to modeling playfulness themselves (Synodi et al., 2015). Teachers’ engagement in playful learning environment was also found to be related to student satisfaction from learning (Kangas et al., 2017). Finally, a longitudinal evaluation of playful curricula found that absence of teacher’s playfulness was often associated with lower levels of child engagement in play and activities in general (Walsh et al., 2011).

Following these studies, we assume that teachers’ playfulness influences preschoolers’ play behavior. The current pilot study addresses the empirical gap in our knowledge on the relation between teachers’ and children’s playfulness. The research hypothesis is that a positive relation will be found between teachers’ and children’s observed playfulness.

## Materials and Methods

### Sample

Thirty-one teacher–child dyads participated in the study. The children were all typically developed and their ages ranged between 40 and 72 months (*M* = 54.47, *SD* = 10.01). Four girls and 27 boys participated [this is due to the nature of the sample, being part of a larger research project that included children with typical development and with autistic spectrum disorder (ASD), which is more common in boys]. The children were all from mid-upper socioeconomic class families. The teachers – all women – were aged 24 to 57 (*M* = 40.97, *SD* = 10.43). All teachers had a degree in education and a teaching certificate. All children learned in urban public preschools and kindergartens supervised by of the Israeli Ministry of Education. All teachers knew the children they were playing with at least 3 months before data collection.

### Measures

#### Background Characteristics

Teachers were asked to report their age and education level. In addition, they were asked to report the child’s age and their length of acquaintance (in months).

#### Developmental Assessment

In order to make sure all children were typically developed, they passed a developmental assessment using the Mullen Scales of Early Learning (MSEL; Mullen, 1995) or the Wechsler Preschool and Primary Scales of Intelligence-Revised (WPPSI-R; Wechsler, 1989), depending on their chronological age. The MSEL is a standardized developmental test for children aged 3 to 68 months, consisting of five subscales: gross motor, fine motor, visual reception, expressive language, and receptive language. It provides separate standard verbal and non-verbal summary mental age (MA) scores. Commonly used and well-validated, the MSEL was translated and standardized in Israel (Ben-Sasson et al., 2007). The WPPSI-R is an intelligence test designed for children aged 2.5 to 7.25 years. It provides verbal, performance, and full-scale IQ scores, converted in the current study into an MA score. It is a well-known measure translated and normed in Israel (Pilowsky et al., 1998).

#### Teachers’ Playfulness

Teachers were asked to complete a background questionnaire as well as the Adult Playfulness Scale (APS; Glynn and Webster, 1992, 1993). The APS is a list of 32 adjectives which are scored on a seven-point scale; five additional facets of adult playfulness may be evaluated (Bozionelos and Bozionelos, 1999; Proyer, 2011). These facets are spontaneous (the alpha-coefficient in this sample was 0.74), expressive (e.g., bouncy vs. staid; α = 0.76), fun (e.g., bright vs. dull; α = 0.75), creative (e.g., imaginative vs. unimaginative; α = 0.78), and silly (e.g., childlike vs. mature; α = 0.79). Glynn and Webster (1992) report satisfactory internal consistencies and test–retest correlations, and a robust factor solution for their instrument. The APS was translated into Hebrew and retranslated for the current study.

#### Children’s Playfulness

The Test of Playfulness (ToP; Bundy, 1997) was used to evaluate children’s playfulness level. The ToP is an observation-based assessment of the playfulness of children between the ages of 6 months and 18 years. It consists of 29 items (e.g., “Engaged in social play”; “Incorporates objects or other people into play in unconventional or variable ways”) scored on three Likert scales: (1) Extent (0 = *rarely or never*, 3 = *almost always*); (2) Intensity (0 = *not*, 3 = *highly*); and (3) Skill (0 = *unskilled*, 3 = *highly skilled*). In our study, one of the items (“Enters a group already engaged in an activity”) was eliminated because it was inappropriate for dyadic play. A complete list of ToP items and examples may be found in Pinchover et al. (2016).

Scoring the ToP utilizes a test-specific keyform, which plots the relative difficulty of each item against the means and standard deviations for all items, and produces a total score ranging from 7 to -7, subsequently translated into a 0–3 score (Bundy et al., 2001).

In addition, Rasch analysis was performed using a “fit-the-model” methodology (Andrich, 1988). Rasch analysis uses a probability model to estimate personal “ability” and item “difficulty” by “comparing the response patterns of individuals to the entire sample” (Duncan et al., 2003, p. 951). Called “logits,” these equal-interval measures reflect the participant’s ability to perform a particular task.

All videotapes were coded by three trained and reliable coders. Inter-coder reliability was established for 15% of the videotapes using inter-class correlations (Koch, 1982), and ranged between 0.70 and 0.79.

### Procedure

As mentioned, the data were collected as part of a larger study that investigated play interactions of children with and without ASD at home and educational settings. The study was approved by the Ethics Committee of the Hebrew University of Jerusalem and the Israel Ministry of Education.

First, the research team contacted teachers in preschools and kindergartens for typically developed children that are supervised by the ministry. Teachers who agreed to participate were asked to write to all the parents in the preschool or kindergarten, explaining about the research and asking their permission for their children to participate. The children who were permitted to participate and who met our age and gender criteria were included in this study. Each teacher was paired to the first child/ two children from her classroom included in the study.

Data were collected during the second and third quarters of the school year, in order to let the teachers get to know the children and establish their relationship before the research. A certified psychologist administered the developmental assessment to each child at their home. Parent who were interested in getting the test result, could receive a formal report and meet with the head psychologist for discussion. Each child–teacher dyad was videotaped in a 30-min play interaction at school, playing “as you usually do.” The dyad was offered to play with developmentally appropriate and attractive toys (e.g., books, blocks, puzzles, dolls, cars) provided by the researchers. Finally, teachers were asked to complete a demographic questionnaire and the APS.

### Data Analysis

First, descriptive statistics and bivariate Pearson’s correlations between research variables were calculated. Next, a mediation model was tested to investigate the indirect link between children’s chronological age (CA) and playfulness as mediated by teachers’ playfulness aspects. The mediation analysis was conducted using PROCESS macro for SPSS (Models 4; Preacher and Hayes, 2008; Hayes, 2009), which enables examination of mediation models on small samples, using the bootstrapping method. The analysis provides bootstrapped confidence intervals (CIs) for the conditional effects; when the model is significant, 0 will not be included in the CI, and the CI will be 95%.

## Results

### Descriptive and Bivariate Statistics

**Table 1** presents means, standard deviations, and inter-correlations for all variables. Research hypotheses were examined using *t*-tests and Pearson correlations. Child’s gender and child’s playfulness as well as teacher’s playfulness existed independently from each other (*U* = 35.00, *p* = 0.35 and *U* = 38.00, *p* = 0.17, respectively). No relationship was found between children’s chronological or mental age (CA/MA) and their level of playfulness (*r* = 1.63, *p* = 0.38 and *r* = 0.05, *p* = 0.78, respectively). However, a negative correlation was found between children’s CA and teachers’ perceived silliness (*r* = 0.41, *p* < 0.05), indicating that teachers of younger children perceived themselves as more “silly.” A positive relation was found between two of the APS subscales and children’s playfulness. Specifically, teachers’ spontaneity and silliness were both positively related to higher levels of child playfulness (*r* = 0.38, *p* < 0.05 and *r* = 0.35, *p* < 0.05, respectively). No significant relation was found with the expressive, fun and creative subscales. A positive, non- significant, correlation was found between the teachers’ overall APS score and child playfulness (*r* = 0.24, *p* = 0.07).

**Table 1:** Descriptive statistics and inter-correlations (*N* = 32).

	*M*	*SD*	1	2	3	4	5	6	7	8	9
(1) Child’s CA (months)	54.47	10.00									
(2) Child’s MA (months)	63.79	16.03	0.932^∗∗^								
(3) Teacher’s age (years)	40.97	10.43	0.188	0.090							
(4) Teacher’s spontaneity	24.90	6.23	-0.152	-0.092	-0.145						
(5) Teacher’s expressiveness	26.53	4.15	-0.089	0.007	-0.245	0.680^∗∗^					
(6) Teacher’s fun	26.84	3.94	0.149	0.218	-0.175	0.334	0.534^∗∗^				
(7) Teacher’s creativity	17.15	2.79	0.078	0.110	0.236	0.164	0.381^∗^	0.798^∗∗^			
(8) Teacher’s silliness	14.03	3.80	-0.413^∗^	-0.315	-0.521^∗∗^	0.481^∗∗^	0.394^∗^	-0.124	-0.304		
(9) Teacher’s total playfulness	109.46	14.84	-0.140	-0.039	-0.277	0.853^∗∗^	0.880^∗∗^	0.674^∗∗^	0.498^∗∗^	0.478^∗∗^	
(10) Child’s playfulness	-2.82	0.89	0.163	0.143	-0.301	0.379^∗^	0.109	-0.003	-0.157	0.335^∗^	0.243

*CA, chronological age; MA, mental age; ^∗^p < 0.05; ^∗∗^p < 0.01.*

### Mediation Model

Given the correlation found between children’s age and teachers’ silliness, as well as between teachers’ silliness and children’s playfulness, a mediation model was run to examine whether the relationship between children’s age (independent variable) and playfulness (dependent variable) was mediated by teachers’ silliness (mediator variable). According to Hayes (2009), a significant association between independent and dependent variables is not necessary for testing and establishing mediation: “a failure to test for indirect effects in the absence of a total effect can lead to you miss some potentially interesting, important, or useful mechanisms by which X exerts some kind of effect on Y” (p. 414). dependent variables is not necessary for testing and establishing mediation, and A significant mediation effect was found (indirect effect = 0.017, *SE* = 0.12; 95% confidence interval: LLCI = -0.054, ULCI = -0.003). Thus, the relationships between a child’s age and playfulness was mediated by teacher’s silliness.

## Discussion

This pilot study is one of the first to demonstrate that aspects of teachers’ playfulness are positively related to higher levels of children’s playfulness. The study showed that teacher spontaneity and silliness were positively related to child playfulness. In addition, the relation between teachers’ overall playfulness and children’s playfulness was close to significant, and should be reexamined on a bigger sample. Those results are some of the first that empirically support the hypothesis that aspects of teachers’ playfulness in ECE, and specifically in teacher–child play interactions, are related to higher level of child playfulness. Note, however that this relation can be bidirectional nature, and that children’s classroom behavior can also affect teachers’ playfulness.

Our findings confirm Lieberman’s (1977) early intuition regarding the importance of teachers’ playful behavior in ECE: a teacher who knows how to act playfully by joking, teasing, clowning, and acting silly playful teacher is more likely to facilitate playful behaviors in her students. Specifically, in the current study, teachers’ spontaneity and silliness were found to be significant in that regard. Spontaneity has a major role in Lieberman’s (1977) definition of playfulness, referring to the individual’s ability to be flexible. Glynn and Webster (1992) described spontaneity as the ability to be free-spirited and less disciplined. It is possible that being more spontaneous allow teachers to concentrate more on play and less on discipline in their classrooms, which in turn gives the children more opportunities to be playful. However, it is also possible that teacher’s spontaneity is affected by the children’s behaviors in class. Further research is needed to fully understand this relationship.

The other teacher behavior found in the present study to be significantly related to child playfulness, silliness – defined as childlike behavior (Glynn and Webster, 1992) – and has not been widely investigated. Jung (2011) found that silly facial expressions play a part in teachers’ playful behaviors that help young children cope with stressful transitions. Cohen (2008) suggested that caregivers use silliness to solve stressful conflicts. Similarly, Kuhaneck et al. (2010) suggested that a therapist who feels free to act silly can more easily engage children in an activity, and that silliness, like other playful behaviors can be acquired. Silliness may therefore help teachers engage children in play and other activities. Another possible to explanation for the relationship between teacher silliness and child playfulness may be that since silliness is a very salient behavior it may be easy for children to copy and incorporate in their own playful behavior.

In the current study, the mediation model showed that teachers’ silliness was higher when children were younger, so that younger children would be more playful given higher levels of teachers’ silliness. No direct correction was found between children’s age and playfulness level. However, it seems that age did have a role in teacher playfulness, so that it was indirectly related to children’s playfulness. This may be explained by the fact that in recent years, play is sidelined by early learning standards and assessment of academic attainments (Roskos and Christie, 2007; Dickey et al., 2016); thus, the older the children are, the less time they have to play in school. However, a Vygotskian approach to play indicates that play and academic development are not mutually exclusive – in fact, scaffolding play can promote not only the development of play itself, but also the acquisition of academic skills (Bodrova, 2008). Preselection can also explain this finding: teachers who perceived themselves as “sillier” chose to work with younger kids with whom they felt more comfortable to expresses this trait. Johnson et al. (2013) argued that “No longer is it enough for an ECE teacher to simply respect playfulness in young children; they must also be playful themselves and master play facilitation techniques” (p. 271). The findings of the current study provide tentative support for this claim.

Knowing the benefits of play and playfulness in ECE (Singer, 2013) and their importance for child development, it is meaningful to address teachers’ understanding of play and playfulness, and to promote their own playfulness in order to enable them to help children express and develop their playfulness. According to Broadhead et al. (2010) it is essential that teachers observe children, especially when they play, so that they can gain the ability to understand and support playful behavior and learning.

Although playfulness is usually considered a personal trait, playful behaviors such as silliness may also be seen as skills that can be acquired and honed. Using silliness, spontaneity and other play behaviors for pedagogical purposes is easier when one has mastered play-based assessment and communication techniques (Jones and Reynolds, 2015). Unfortunately, however, play and playfulness are often neglected in teacher education (Johnson et al., 2013).

Carter (1993) suggests three stages in training teachers to be more playful, including aspects highlighted in the current research. First, the teachers need to identify their experience and attitude toward play. Next, they need to be taught to pay attention and understand children’s play. When these two skills are attained, they will be able to move to the third stage of training, which includes various playful activities allowing teachers to practice playful behavior. Similarly, Jones and Reynolds (2015) suggest that practice and exercises in remembering one’s own past play can help teachers stay in touch with the child inside them, which will help them act more playfully. Trawick-Smith and Dziurgot (2010) showed that teachers who had better education were more likely to perform good-fit play interactions. Teacher training programs should reconsider how to expand teachers’ knowledge about and understanding of play and playfulness and how they might develop their own playfulness – information that is unfortunately lacking in current early-years teacher training (Jung and Jin, 2015).

The current study is a pilot study, and despite its interesting findings, its limitations must be taken into consideration. First, its sample is small and not fully representative. For example, all the teachers in the sample were female. Although ECE teachers in Israel are females, it is important to include male teachers in research and to investigate gender differences. Future research should continue this investigation using larger and more representative samples. Second, longitudinal research should be conducted in order to determine causality. Third, teachers’ playfulness was measured using a self-report questionnaire, since, to the best of our knowledge, no observational measurement for adult playfulness exists. However, in order to gain deeper understanding of how teachers use playful behaviors in play interaction in ECE, observational measurement should be considered. Furthermore, all teachers have complete the APS *after* the play-interaction had been videotaped. The play interaction could thus have affected the way they completed the survey. Finally, despite its advantages, the APS, was criticized for poor theoretical background, psychometrics and validity. Therefore, future research should consider using alternative measurements of adult playfulness (such as provided by Barnett, 2007; Shen et al., 2014; Proyer, 2017). In addition, the current research uses new translation of the APS scale that has been developed for this study. The validity of the Hebrew translation of the APS needs further testing.

In addition, further research should investigate additional child and teacher characteristics that can be related to those findings, for example, by looking into the variability among teachers in their play understanding and practices. Promoting teachers’ playfulness and playful ECE environment can eventually lead to better classrooms and play experiences for children. As part of a broader research project that investigated play interactions between and children adults at home and educational settings, it will be interesting to look at differentness in playfulness between parent and teachers and its relations to child’s playfulness. Finally, in is important to remember that one-on-one teacher–child play interactions are not common in today’s ECE settings. Future studies can examine group play interactions with teachers, as well as whether improved teacher–child play interactions also lead to improved child–child play and other interactions.

## Author Contributions

The author was responsible for research design, data collection, data analysis, and writing the paper.

## Conflict of Interest Statement

The author declares that the research was conducted in the absence of any commercial or financial relationships that could be construed as a potential conflict of interest.
